# Small Duct Primary Sclerosing Cholangitis in Association With Hepatitis C Virus Infection: A Case Report

**DOI:** 10.4021/gr282w

**Published:** 2011-01-20

**Authors:** Suresh Kumar Nayudu, Kavitha Kumbum, Bhavna Balar, Masooma Niazi, Sridhar Chilimuri

**Affiliations:** a Department of Medicine, Bronx Lebanon Hospital Center, Affiliated to Albert Einstein College of Medicine, Bronx, NY, USA; b Division of Gastroenterology, Bronx Lebanon Hospital Center, Affiliated to Albert Einstein College of Medicine, Bronx, NY, USA; c Department of Pathology, Bronx Lebanon Hospital Center, Affiliated to Albert Einstein College of Medicine, Bronx, NY, USA

**Keywords:** Primary sclerosing cholangitis, Small duct, Hepatitis C virus

## Abstract

Small duct primary sclerosing cholangitis (PSC) is characterized by cholestatic liver function tests, histological evidence of PSC but absence of classic cholangiographic findings. Large duct or classic PSC in association with hepatitis C virus (HCV) infection has rarely been reported. However to the best of our knowledge small duct PSC in association with HCV infection has not been reported. We report this case of small duct PSC in a patient with HCV infection. HCV infection in our patient was successfully treated with ribavirin and peg interferon alfa-2a, as evidenced by undetectable HCV ribonucleic acid levels. However, the patient had persistently elevated liver function tests suggestive of cholestasis. Endoscopic retrograde cholangiopancreatography (ERCP) revealed normal architecture of bile ducts. Hence patient underwent liver biopsy and its histopahological findings were suggestive of PSC. He had colonoscopy along with biopsy and inflammatory bowel disease (IBD) was ruled out.

## Introduction

Small duct primary sclerosing cholangitis (PSC) is a rare disorder characterized by cholestatic liver function tests, histological evidence of PSC but without classic endoscopic retrograde cholangiopancreatography (ERCP) findings. Inflammatory bowel disease has been associated with large duct as well as small duct PSC, although the small duct variant is rare. Association of PSC with hepatitis C virus (HCV) infection is very rare. In fact small duct PSC in association with HCV infection has not been reported. We present a case of small duct PSC in a patient with HCV infection who has been successfully treated but had persistently elevated liver function tests.

## Case Report

A 26-year-old man presented to the emergency department with severe upper abdominal pain of two days duration. He denied any fever, nausea, vomiting or gastrointestinal bleeding. His medical history included genotype 1a HCV infection diagnosed 5 years ago. There were no clear risk factors for HCV infection. He was treated successfully with Ribavirin and Peg interferon alfa-2a for 48 weeks. At the end of the treatment his HCV RNA levels were undetectable. However, his alanine amino transferase (ALT), aspartate amino transferase (AST), and alkaline phosphatase levels remained high after the completion of the treatment. His surgical history included right inguinal hernia repair 5 years ago and elective laparoscopic cholecytectomy 3 years ago for cholelithiasis. He denied alcohol or substance use.

Initial physical examination revealed mild tenderness in both upper quadrants of the abdomen without guarding or rigidity. He was afebrile and hemodynamically stable. His amylase, lipase, total and direct bilirubin levels were elevated. He underwent computerized tomography (CT) of the abdomen which showed findings suggestive of acute pancreatitis. Ultrasonography of the abdomen was performed which showed mildly dilated common bile duct. He was admitted to the hospital with the impression of acute pancreatitis. He underwent ERCP for evaluation of dilated bile duct that revealed biliary sludge with normal architecture of bile ducts. A stent was placed during ERCP and his clinical condition improved gradually. His amylase, lipase and bilirubin levels normalized, but ALT, AST and alkaline phosphatase levels remained persistently elevated. He was discharged with follow-up appointment in gastroenterology clinic.

Further workup including serum ceruloplasmin, copper and iron levels were normal. Screening for hepatitis A virus and hepatitis B virus was negative. He was tested negative for rheumatoid factor, anti-nuclear, anti-smooth muscle, anti-mitochondrial, anti-myeloperoxidase and anti-proteinase-3 antibodies. His rapid plasma reagin and human immunodeficiency virus tests were negative. Patient underwent CT guided needle biopsy of the liver.

**Figure 1:**
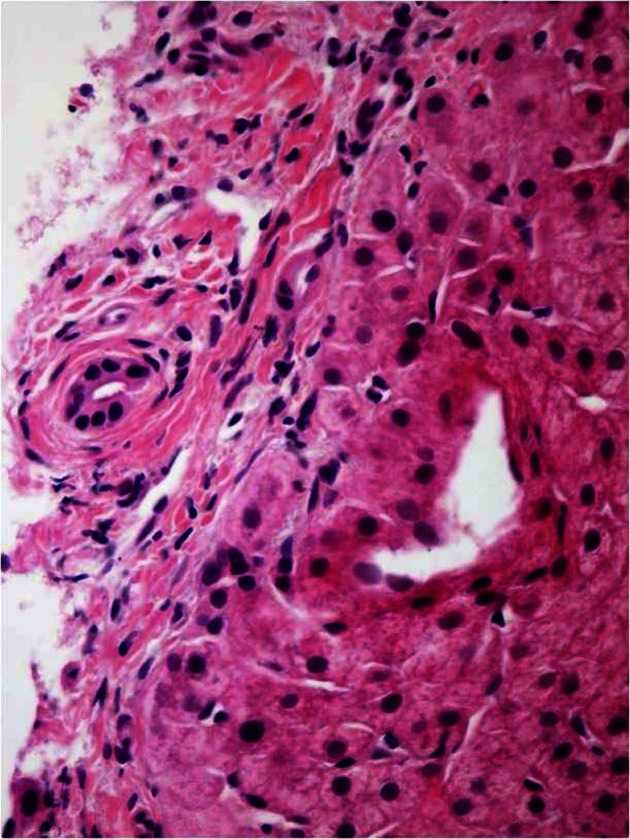
Liver biopsy showed “Onion-skin” periductal fibrosis.

**Figure 2:**
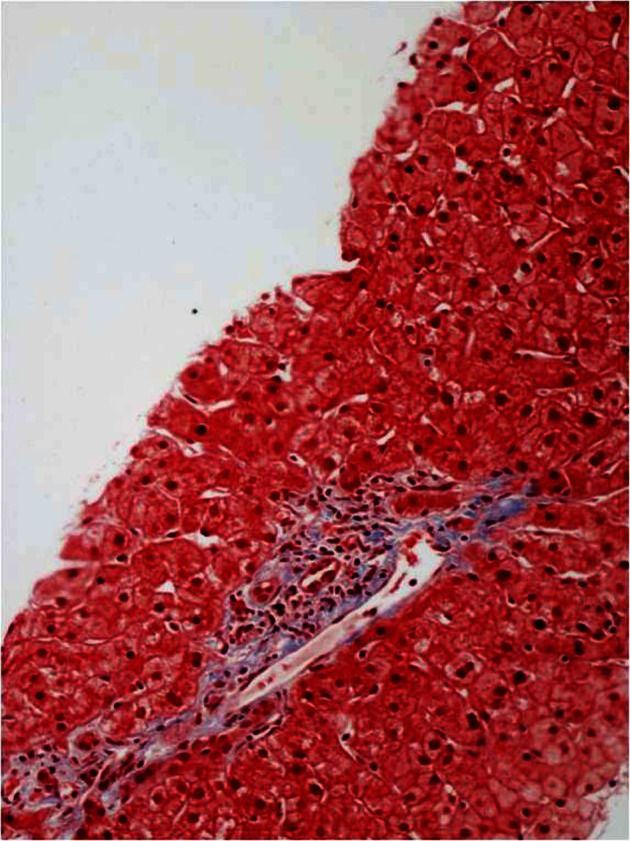
Liver biopsy showed bile duct loss and ductular reaction.

**Figure 3:**
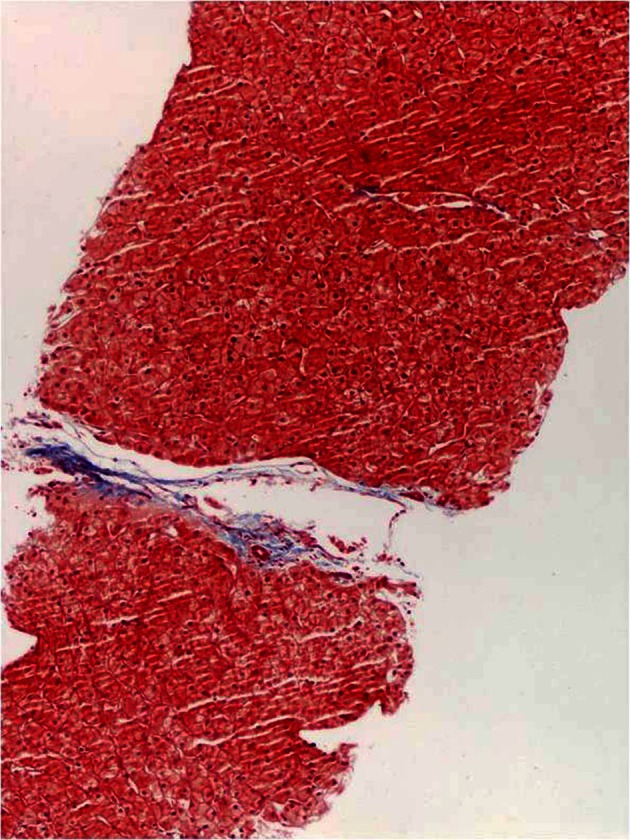
Liver biopsy showed hypocellular fibrous septa.

Liver biopsy showed: “Onion-skin” periductal fibrosis (Fig. 1), bile duct loss, ductular reaction (Fig. 2), hypocellular fibrous septa (Fig. 3) and possible bile duct scar which were suggestive of PSC. Subsequently the patient underwent colonoscopy to rule out inflammatory bowel disease. The histopathological findings of colon and terminal ileal specimens did not show any evidence of inflammatory bowel disease. At this point he remains clinically asymptomatic and does not require any treatment.

## Discussion

Small duct PSC previously known as pericholangitis [1, 2] is described as having biochemical evidence of cholestasis, histological features of PSC but with normal ERCP findings [3]. It has been known that small duct PSC occurs in association with inflammatory bowel disease [1, 4, 5], esinophilic gastroenteritis [6], as a component of overlap syndrome with autoimmune hepatitis [7, 8] and in explanted liver [6]. Both small duct and large duct PSC resemble in clinical, biochemical and histological features [4]. However, small duct PSC occurs less frequently compared to large duct PSC [9], affecting adults more than children [8], males more than females [4]. Natural history of small duct PSC is more benign compared to large duct PSC [10, 11]. Significant proportion of small duct PSC patients may progress to large duct PSC [1, 10, 12] or end stage liver disease requiring liver transplantation [9]. Progression to cholangiocarcinoma never occurs in small duct PSC patients compared to large duct PSC patients [9, 12, 13]. Small duct PSC patients live longer without transplantation [9, 10] and it may recur after transplantation [10].

Classic or large duct PSC in association with HCV infection is very rare [14]. There have been two case reports describing the association; the first case was reported in a patient with HCV infection after successful treatment with interferon and second in a patient where interferon therapy could not clear the virus. However, currently there is no available data on association of small duct PSC with HCV infection. As the available evidence suggests that small duct PSC may represent earlier spectrum of disease progression [1, 9, 10, 12, 13], this case may represent onset of disease process and it may progress to large duct PSC in future.

Abnormal liver function tests always mandate work up to understand and diagnose disease process. This case signifies the importance of pursuing further noninvasive and invasive interventions to achieve the diagnosis in patients with biochemical evidence of cholestasis. Before attempting invasive interventions other causes of liver function abnormalities including infectious, metabolic and autoimmune [7, 8] etiologies have to be ruled out. Invasive procedures like ERCP, liver biopsy and colonoscopy to rule out coexisting bowel disease [4, 5] may be needed to confirm the diagnosis.

This case warrants further studies to understand pathogenesis and treatment modalities of small duct PSC particularly in association with HCV.
